# Composition and Structure of a Large Online Social Network in the Netherlands

**DOI:** 10.1371/journal.pone.0034760

**Published:** 2012-04-16

**Authors:** Rense Corten

**Affiliations:** Department of Sociology/ICS, Utrecht University, Utrecht, The Netherlands; Hungarian Academy of Sciences, Hungary

## Abstract

Limitations in data collection have long been an obstacle in research on friendship networks. Most earlier studies use either a sample of ego-networks, or complete network data on a relatively small group (e.g., a single organization). The rise of online social networking services such as Friendster and Facebook, however, provides researchers with opportunities to study friendship networks on a much larger scale. This study uses complete network data from Hyves, a popular online social networking service in the Netherlands, comprising over eight million members and over 400 million online friendship relations. In the first study of its kind for the Netherlands, I examine the structure of this network in terms of the degree distribution, characteristic path length, clustering, and degree assortativity. Results indicate that this network shares features of other large complex networks, but also deviates in other respects. In addition, a comparison with other online social networks shows that these networks show remarkable similarities.

## Introduction

For social scientists, the remarkable rise to prominence of online social networks is relevant for at least two reasons. From a substantive point of view, online social networks provide a novel way of social interaction, providing individuals with new ways to communicate, spread information, and coordinate collective action. In addition, online social networks may be interesting from a methodological perspective, as data from such networks provides us with new ways to study social structure and provide a way out from the problems of traditional social networks research, as will be outlined below.

I adopt the common definition of Online Social Networks as “as web-based services that allow individuals to (1) construct a public or semi-public profile within a bounded system, (2) articulate a list of other users with whom they share a connection, and (3) view and traverse their list of connections and those made by others within the system” [1]. In addition, I focus in this paper on online *friendship* networks, which can be defined as Online Social Networks in which social interaction *per se* is the main focus of the service. *Facebook* [2] is the prime example of an online friendship network. Services like *Flickr* [3], which are primarily centered around displaying photos and have “social” features in addition to that are not included this definition.

The availability of online social network data holds the promise of a way out from some common methodological problems in “traditional” survey-based research on social networks. Such survey research falls generally in one of two categories. In the first category, a sample of individuals is surveyed about their personal social networks, resulting in a dataset of “ego-networks” [4]. In the second category, the researcher defines a social group within some substantive bounds (such as a local community or an organization), and surveys the members about *all* their contacts within this group. This approach may be labeled as the “sociometric approach” [5].

The advantage of the ego-networks approach is that one can, via appropriate representative samples, study personal social networks in relatively large populations. The disadvantage, however, is that one can not study network structure beyond these personals networks. Questions about population-level properties of network structure such as characteristic path length or community structure cannot be measured with this approach. The sociometric approach, on the other hand, does allow for studying such structural questions, but is in practice limited to relatively small groups and can not be applied to large populations. The result of these limitations is that, despite the accumulation of a vast body of literature on social networks in the past decades, we still know very little about social network structures of entire societies, even though society as a whole is arguably sociology's natural unit of analysis.

Online social network data have the potential to alleviate these limitations by combining the “best of both worlds.” On the one hand, because data collection is effectively automated, online network data can be collected on very large groups. On the other hand, because one is not restricted to using random samples, one can collect information on *all* relations within the given population, which allows to study social structure. An additional advantage of data from online social networks is that they provide actual measures of behavior (members choosing their “friends”) as opposed to answers to survey questions, thereby circumventing problems of recall, practical limits to survey length, interviewer effects, etc.

However, research on online networks has only rarely fulfilled these promises. Although massive amounts of data are automatically collected by service providers, these data are only very rarely available to researchers. Consequently, many studies are based on samples [6], [7], typically collected through variations of snowball sampling. However, such samples are likely to be biased in one way or another [8].

In rare cases, researchers have been able to analyze complete datasets, such as the *MSN Messenger* [9] network (which is not an online social network as defined above) and *Cyworld* [10], or unbiased samples [8]. Results from these studies often differ from what is typically found in studies based on samples and studies of other types of complex networks. Specifically, it is found that online friendship networks do not show the power-law degree distributions typically found in large networks. Hence, we have reason to wonder if there is something special about these friendship networks that makes them behave differently.

In this paper, I use a dataset obtained from the Dutch service *Hyves* [11] that does not suffer from sampling problems, and in addition, contains information on a online networking service that is very popular in a single country, and has a relatively large proportion of the population as members. I provide a first explorative analysis of the structure of this network.

The contribution of this paper to the literature is twofold. First, this study is, to my knowledge, the largest study of an online social friendship network in which the population of the online social network coincides to a large extent with the population of a well-defined society (i.e., the Netherlands), which allows me to explore the extent to which the online social network can be interpreted as a measure of the friendship network within this society. Earlier studies have either focused on online social networks that span multiple societies [8] or on online social networks that cover a much smaller portion of the population [10].

If one intends to use data from online social networks to study social relations in a society at there are a number of reasons why the Netherlands, and Hyves in particular, might be an especially interesting case to study. First, the availability of internet access in the Netherlands is among the highest in the world [12], [13]. Second, the use of online social networks is wide-spread. Survey show that report that 80% of people aged 16–35 in the Netherlands use online social networks on a monthly basis, while 45% claims to use them daily [13]. Third, the membership of Hyves is very large as compared to the size of the general population. At the time of writing, Hyves had more than 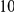 million members, while the size of the Dutch population was about 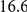 million, thus potentially covering more than 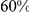 of the population. As a point of reference, the Cyworld network studied by [10] potentially covered 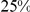 of the South Korean population.

The second contribution of the paper is to make an implicit comparison between *Hyves* and two other online (friendship) networks for which comparable data (i.e., complete snapshots or unbiased samples) are available, namely *Facebook* and *Cyworld*. I find that, despite considerable differences in size and population between these networks, some of their structural features are strikingly similar.

Thus, I aim to answer the following questions. First, what is the composition of the Hyves network in terms of the demographic characteristics of its members? An answer to this question will help to assess to what extent research findings on this network can be generalized to the general population. Second, what characterizes the structure of this network? I assess the structure of the network in terms of fundamental properties like the degree distribution, clustering, characteristic path length, and degree assortativity. Third, how does the Hyves network compare to other online friendship networks in terms of structure?

### Related literature

While social network analysis has a long history in sociology [14], [15], the rise of the internet and the increasing availability of large datasets have in recent decades sparked interest from other disciplines including (statistical) physics, economics, and computer science. Much of this literature, which has developed quite independently from the existing sociological literature, focusses on the structure and dynamics of large complex networks, that may or may not be social networks. The volume by [16] provides a good overview of this literature. The majority of research on online social networks falls within this relatively young tradition of “network science” [17].

A central issue in the literature on complex networks has been the so-called *small-world problem*, that is, the observation that although social networks are typically very clustered – most of your friends are also friends of each other – they also have a short characteristic path length, such that many nodes in the network can be reached in surprisingly few steps [18]. In a seminal paper, [19] show that this phenomenon occurs not only in social networks but also in many other types of networks, and can be explained as the interplay of structure and randomness. Furthermore, they argue that the presence of small-world properties potentially has important consequences for the spread of infectious diseases and the adoption of cooperative behavior.

A second area of interest in the study of large networks has been the shape of *degree distributions*, that is, the statistical distributions of the number of nodes' connections. In a number of influential papers [20], [21], it has been argued that many large networks, including social networks, are characterized by *scale-free* or *power law* degree distributions, that is, degree distributions in which the ratio of high-degree nodes and low-degree nodes is constant over the range of degrees. A typical feature of such distributions is the presence of a “fat tail,” such that the occurrence of nodes with extremely high degrees is relatively likely. Power-law distributions have been argued to exist in networks as diverse as power grids, genetic networks, the internet, film actor collaborations, and indeed online networks [6], [7], [22], [23].

However, more recently, this view has been challenged by findings that show that in many cases, power law distributions actually fit the data poorly [24] and that in the case of social networks, other distributions often provide a better fit. Jackson and Rogers [25] propose a “hybrid” model for growing networks that parametrizes the balance between purely random growth and “network-based” network formation in the sense that new nodes are more likely to connect to existing nodes that already posses many links (e.g., preferential attachment [20]). The combination of these two processes generates degree distributions that fit many empirical distributions well. In the case of online *friendship* networks, the few studies that used complete or unbiased data indicate that these networks do *not* have power-law degree distributions, but instead show “multi-scaling behavior,” suggesting the presence of different types of nodes [8]–[10]. These findings suggest that, contrary to the view that practically all large complex networks are governed by “generic topological and dynamical principles” [21], online networks that consist of *social* interaction may behave in in ways that significantly different from other large systems.

A common observation in empirical research on large networks is the existence of a *giant component*, that is, a connected subset of the network that contains the large majority or even all of the nodes [26]. The emergence of such a giant component is a classic result of random graph theory [27], and is therefore not very surprising. Nonetheless, [28] report that some online social networks show a non-trivial component structure in that, besides a giant component and a large number of isolated nodes, they also contain a “middle region” small components that are predominantly start-shaped.

Finally, social networks are often found to be *homophilous*, in the sense that nodes with similar characteristics are more likely to be connected than dissimilar nodes [29]. A particular form of homophily is *degree assortativity*, that is, the tendency of high-degree nodes to be connected to other high-degree nodes, and of low-degree nodes to be connected to low-degree nodes. Degree assortativity is indicative of (but does not prove) a so-called core-periphery structure, in which there exists a core of high-degree nodes who are relatively well connected to each other, and a periphery of lower-degree nodes [30]. This phenomenon is typical for social networks as opposed to technological networks (with some exceptions [31]) [30], and has also been observed in online social networks [8], [10].

## Materials and Methods

**Figure 1:**
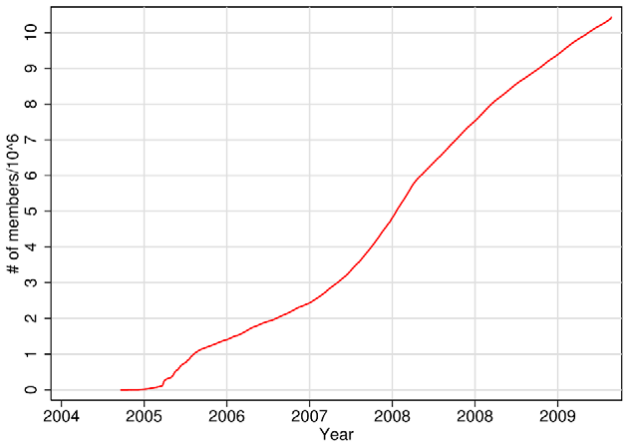
Growth of the Hyves network.

The data for this study were provided by the online social network service Hyves (in the remainder of the paper, I use the name “Hyves” to refer to both the online social network service and the company that provides the service). Hyves, based in Amsterdam, has been active in the Netherlands since 2004 though its website www.hyves.nl. Almost since its inception, the service has seen fast growth and has received considerable attention in the popular media. Figure 1 depicts the growth of the number of members over time. Despite strong competition from other services, in particular Facebook, Hyves was at the time of writing still the leading online social network service in the Netherlands [32].

The service allows users to create an online profile on which they can share personal information and photos, maintain a blog, and advertise goods and services, among other activities. Most importantly, users maintain a list of *Friends* on their profile pages (following [1], I use the capitalized “Friend” and its derivatives to refer to social relations in the sense of connections in online social networks). One can become Friends with another member by sending a “Friendship request,” which the other member will have to confirm. Thus, Friendship is always reciprocal. Friends can interact by leaving messages (“scraps”) on each other's profile pages, via live chat, and other means. Members have the ability to determine whether the information on their profile is completely public, only visible to members of Hyves, or only visible to Friends, which these settings can often be different for different types of information. Profiles in principle remain active until they are explicitly removed by the respective member. In its basic functionality, Hyves is rather similar to the US-based service *Facebook*. While Hyves is mainly a Dutch service, the website is also available in English.

The dataset used in this study consists of an anonymized snapshot of the entire network provided by Hyves.nl in July 2010 for the purpose of this study. A node in this network is an individual member's profile page; edges are Friendships between these profiles. Although it is technically possible for an individual person to maintain multiple profiles on Hyves, I do not expect this to be a wide-spread phenomenon because it is impractical (e.g., logging into different accounts simultaneously is difficult) and does not serve the main purpose of Hyves. In addition, the data contain for each member the date of joining the network, and if the member provided this information, his or her gender, age, and place of residence. It should be noted that data collection for this study did not require active participation by the members of Hyves; the data are merely (anonymized) digital records of previously made decisions. As such, the data can be considered equivalent to “archival” records. Utrecht University regulations do not require explicit approval by an ethics committee for studies that do not involve a medical component.

As customary in online social friendship networks, regular Friendships in Hyves require the consent of both members involved, and are thus symmetric. In addition, Hyves also allows for asymmetric (“one-way”) Friendships to certain celebrity members. Although such asymmetric Friendships comprise only about 4% of all Friendships, I focus in this paper only on the symmetric Friendships and remove the asymmetric Friendships from the data.

Because the data contained no directed measure of nationality and automatic coding of place of residence to country of residence proved unfeasible, I drew a uniform random sample of 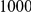 nodes and hand-coded the data on place of residence to country of residence.

The age variable contained a number of unlikely high values and showed some suspicious clustering around high values, especially for values higher than 97. Therefore, I discard all values above 97.

A number of measures are of interest when studying the structure of the network. The *degree* of a node denotes the number of Friends of a node. The shape of the distribution of node degrees provides a first characterization of the structure of a network. A common finding on large networks is that degree distributions follow a power law. A power law distribution may be graphically identified as a straight line in a plot with log-log scales, but can also be statistically fitted using maximum likelihood estimation [24].

According to the hybrid model by Jackson and Rogers, the cumulative degree distribution is given by
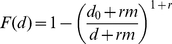
(1)in which 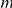 is half of the average degree, 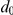 is the minimal degree (set to 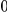 in our case), and 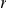 represents the relative importance of random link formation and preferential attachment. 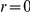 represents pure preferential attachment and 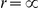 represents a completely random process. I estimate 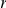 using the iterative regression procedure described in [25]. In addition, I also fit several other common models for skewed distributions, in particular the log-normal distribution and the stretched exponential distribution, using maximum likelihood methods.

Clustering of social relations (also know as *transitivity* in the social networks literature) can be assessed via the *clustering coefficient*, which is defined as the proportion of the pairs of Friends of a node who are also Friends of each other. To obtain a measure of clustering for the network as a whole, I simply compute the average clustering coefficient.

A *component* of the network is a subset of nodes that are directly or indirectly connected.

The *distance* between two nodes denotes the shortest path between those nodes. A typical measure of characteristic path length is average distance, but the computation of average distance for very large networks is usually infeasible. Instead, I look at the *effective diameter*, which can be defined as the smallest number of steps in the network at which at least 90% of all connected pairs of nodes can be reached, but “smoothed” to allow for non-integer values [33], [34].

*Degree assortativity* captures the extent to which high-degree nodes tend to be connected to other high-degree nodes, and low-degree nodes to low-degree nodes. I measure degree assortativity here as the *degree correlation*, expressed as the Pearson correlation coefficient of the degrees associated with the nodes found at either side of the edges [31]. For computational reasons, I compute the degree correlation on a sample of the edges.

For the comparison between Hyves and other online social networks, I rely on two sets of results reported by other researchers on the online social networks Facebook and Cyworld. The choice for these two cases is motivated by two restrictions. First, I want to compare Hyves to other online social networks that were designed for the same purpose, that is, as a generic platform to maintain social relations. Online social networks that are tailored to more specific forms of social interaction, such as dating websites, fall outside this scope. Arguably, the specific purposes of such networks make their dynamics potentially so different that a meaningful comparison is difficult. Second, given the first restriction, we need cases for comparison for which published results are available that are unlikely to suffer from data collection biases as described above.

To my knowledge, Facebook and Cyworld are currently the only two cases that meet both restrictions. Gjoka et al. [8] studied a random sample of members of Facebook, collecting information on degrees and clustering for the sampled nodes. As the data from this study are publicly available, I use these data to make an explicit comparison between the degree distributions of Facebook and Hyves. Ahn et al. [10] studied the South-Korean network Cyworld, using complete data obtained directly from the service itself, as in the current paper. Because the data of that study are not public, I rely on the results as reported in their article.

## Results

### Composition

Table 1 shows summary statistics for a number of individual-level variables. A first result to notice is that of Hyves' 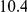 million members, a substantial fraction have no Friends at all, a phenomenon which has also been observed in other online social networks [35]. For that reason, I also report the mean degree for members who have at least one Friend. Among those, the average degree is about 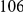. Moreover, while online social networking is sometimes portrayed as a typical teenage activity, I note that the average age is well above that. In terms of gender, males and females are about equally represented among the members. Finally, overall, 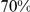 of the members report their city of residence. Of those, I estimate that 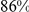 live in the Netherlands. Members living elsewhere are dispersed among many countries, which is illustrated by the fact that the country with the second largest representation is Peru, with an estimated 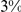 of the members.

**Table 1:** Summary statistics of individual characteristics of Hyves' members.

Variable	Valid 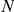	Mean	Std. dev.
Degree	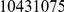	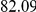	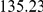
Degree 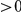	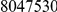	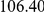	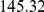
Age	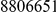	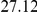	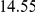
Male	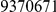	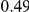	–
Lives in NL	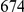	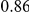	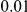

“Valid 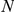
” differs between variables because not all members provide complete information. “Male” and “Lives in NL” are binary variables with 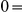
“no” and 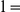
“yes.” The mean of “Lives in NL” is estimated from a hand-coded sample, with the standard error of the estimate reported in the column “Std. dev.”.

**Figure 2:**
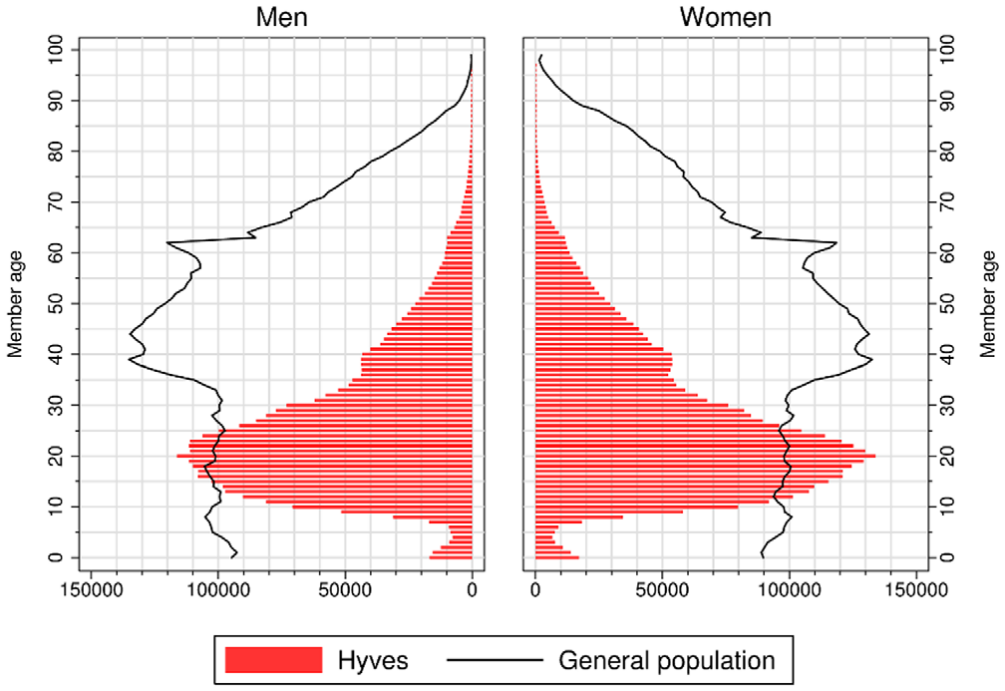
Age-Gender distributions, Hyves and the general Dutch population, 2010 source for population data: [**40**].

To examine to what extent the membership of Hyves is a representation of the Dutch population in terms of its demographic composition, I compare its composition to that of the general Dutch population in Figure 2 in 2010. The bars in this figure represent the members of Hyves, while the lines represent the Dutch population. Note that the horizontal axes show absolute numbers.

A first result that is obvious from the figure is that young people, especially in the age group of 10–25 years, are clearly overrepresented in Hyves as compared to other age groups (in contrast to Facebook, Hyves allows members younger than 13 (with parental consent). The profiles of very young children that are also visible in the figure are most likely started by their parents, for example for the purpose of sharing pictures of newborn children with family and friends). Second, I note that in these age groups, Hyves has more members than there are people in the population. Hypothetically, this may have several causes: it is possible that members misrepresent their age or gender on their profiles, that some members have more than one profile, or that the excess numbers represent members outside the Netherlands. While I cannot exclude the first two possibilities, closer analysis reveals that the proportion of members outside the Netherlands in these age groups (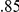 and 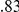 for males and females, respectively) in fact accounts for the difference.

### Structure

**Figure 3:**
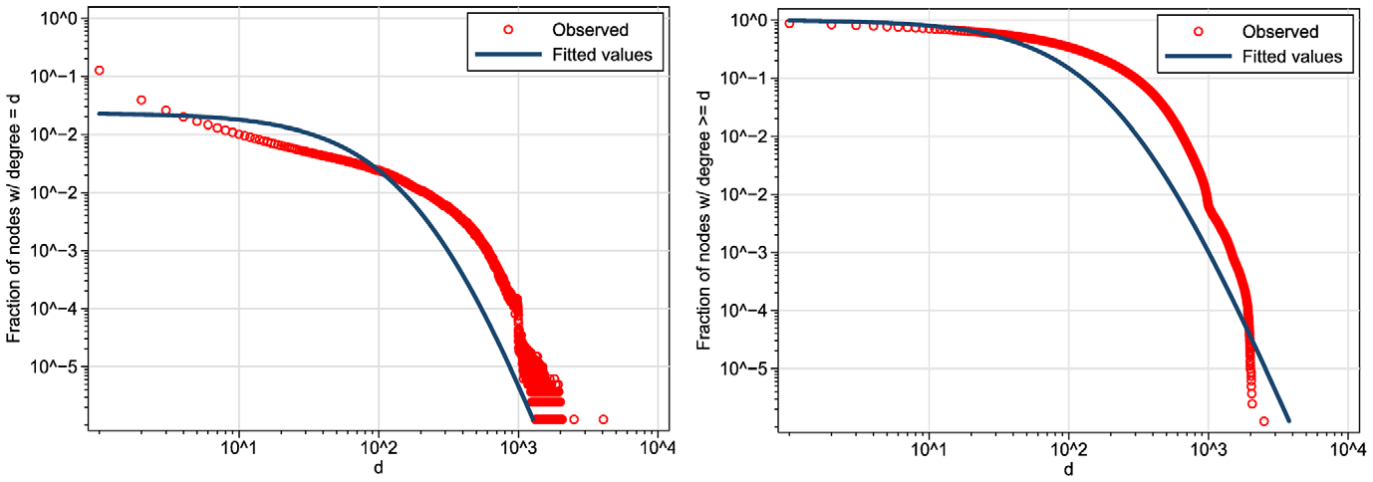
Degree distribution (left-hand panel) and complementary cumulative degree distribution (right-hand panel) of the *Hyves* network.

I begin the analysis of the structure of the network by plotting the degree distribution in Figure 3 (note that the scales of the axes are logarithmic). From the figure it is clear that the distribution is fat-tailed, in the sense that nodes with extremely high degrees are observed relatively often. However, the shape of the distribution is decidedly *not* a straight line, which would be the shape we would expect if the distribution were governed by a power law. At best, the part of the distribution above degree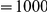 seems to approximate a power law degree distribution. This suggest the presence of two different regimes in the distribution ([10], cf.), an issue to which I will return in the Discussion section.

Next, I fit the hybrid model of Jackson and Rogers [25] to the degree distribution, and find that 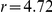, which indicates that the degree distribution is approximated by a process in which random network formation is considerably more important than “network based” network formation. As benchmarks for comparison, we may consider that Jackson and Rogers reported 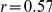 for the World Wide Web, 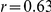 for a citations network, 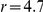 for a co-authorship network, and 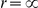 for a network of prison friendships ([25], Table 1). Thus, the estimated value of 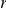 situates the Hyves network in the range of other “social” networks, being close to the value for the citation network.

The variance (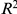) explained by this model is only 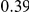 (the 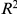 was computed by regressing the observed values on the fitted values). A 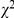 test moreover shows that the fitted distribution deviates significantly from the observed distribution (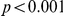). The fitted values from this model are plotted in Figure 3 together with the observed values. While the shape of the fitted distribution is somewhat similar to that of the observed distribution, it is also clear that the model underestimates the occurrence of nodes with small degrees, and somewhat overestimates the occurrence of nodes with high degrees. For comparison, I also fitted log-normal and stretched exponential distributions to the data. The results of these analyses show that also these two distributions deviate significantly from the observed distribution.The parameters of the fitted log-normal distribution are 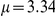 and 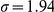; the equation of the fitted stretched exponential distribution is 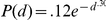. Both distributions deviate significantly from the observed distribution with 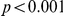 according to a 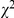 goodness-of-fit test. Thus, although the fit of the Jackson-Rogers model is far from perfect for these data, I nevertheless choose this model for further comparison of Hyves with other online networks (below) because it is at least theoretically founded and is not clearly outperformed by other obvious candidates.

Table 2 summarizes the further structural properties of the network as introduced above. To begin with, we see that although there are many components in the network, almost all the nodes are connected in a single giant component. Thus, in contrast to earlier findings [28], I do not find evidence for a sizable “middle region” of isolated communities. However, I do find that there are numerous small components consisting of up to a hundred members, which I think is still a non-trivial component size. Closer inspection of these smaller components reveals that they are, on average, considerably more homogeneous with respect to age, gender, and geographical location of their members than the giant component. Many, although certainly not all, of the smaller components consist mainly of members outside the Netherlands. In addition, I find that a large majority of these components is star-shaped, in line with [28]. It is an interesting question for further research how these communities emerge and why they are not connected to the rest of the network.

**Table 2:** Structural properties of the Hyves network.

Fitted 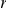	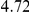
Number of components 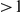	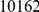
% nodes in largest component	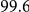
Average clustering	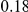
Effective diameter	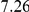
Degree assortativity	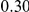

For the Hyves network to display the properties of a “small world,” we would have to observe both clustering and a short characteristic path length. In a either a random Poisson graph or a randomly growing network with the density of the observed network, the expected average clustering would be virtually zero [19], [25]. In contrast, we observe an average clustering of 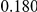, and we can thus conclude that the network is significantly clustered. At the same time, we see that the effective diameter is about seven. Taken together, these results indicate that the Hyves network indeed constitutes a “small world.”

**Figure 4:**
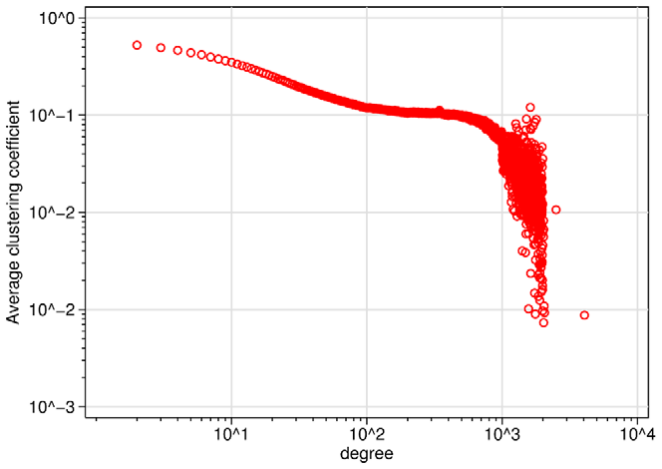
Average clustering by node degree in Hyves.

Figure 4 shows average clustering per node degree. From the figure it is clear that clustering declines with degree, which is a common finding in large networks. However, this relation appears to consist of two regimes: until degree 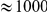, average clustering decreases more or less linearly (on log-log scales), but then the slope changes direction and decreases much more steeply. This finding is similar to the relation between degree and clustering found for Cyworld [10].

To study the degree assortativity of the network, I take a random sample of 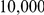 edges. Assortativity is then measured as the Pearson correlation coefficient between the degrees of the nodes at either side of each edge. I find a clearly positive correlation of 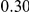, indicating that there is a tendency for high-degree members are to be connected to other high-degree members, and for low-degree members to other low-degree members. This property is associated with the presence of a well-connected core in the network.

### Hyves compared to other OSNs

In this section I compare the structural properties of the Hyves network to those of two closely related online social networks: *Facebook* and *Cyworld*. I chose these two particular networks because for these networks there are published results available [8], [10], that are unlikely to suffer from data collection biases.

**Figure 5:**
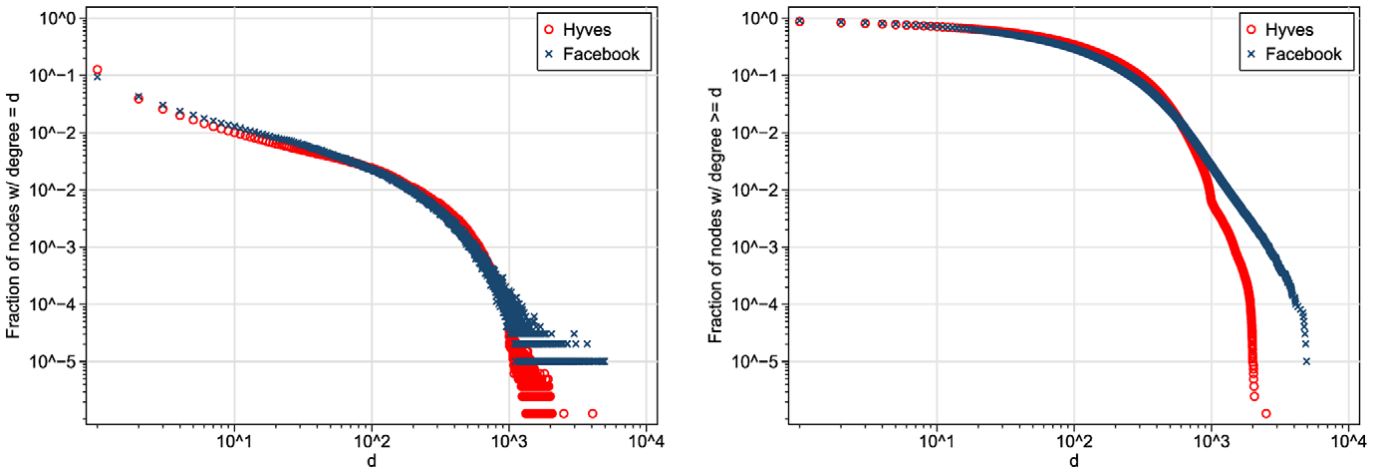
Degree distributions (left-hand panel) and complementary cumulative degree distributions (right-hand panel) of Hyves and Facebook (Facebook source: [**8**]).

Figure 5 shows the (proportional) degree distributions of Hyves and Facebook on the same plot. Visually, the two distributions are remarkably similar. The Facebook distribution, however, does not show the characteristic second “bend” that occurs in the Hyves distribution around 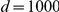, clearly visible in de CCDF. When I fit the Jackson-Rogers model to the Facebook degree distribution, I find that 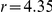, which is rather close to the value I found earlier for Hyves.

Although I cannot reproduce the degree distribution reported for Cyworld [10] here, I note that also the degree distribution of Cyworld looks very similar to those of Hyves and Facebook, and in fact does show the “second bend” that we observe in Hyves.

In Table 3, I compare structural characteristics of Hyves with those of Hyves and Facebook. I find that while the average degrees of Hyves and Facebook are in a similar range, the average degree of Cyworld is much lower, which might be explained by the fact that Cyworld was relatively young at the time of study [10]. The clustering coefficients of the three networks are also very similar, although Hyves is slightly more clustered. Finally, when comparing degree assortativity, I find that Facdebook and Hyves are again similar, although, Hyves appears to be somewhat more assortative. The assortativity coefficient for Cyworld, however,is virtually zero [10]. A possible explanation for this anomalous result could be that the assortativity coefficient, being a Pearson correlation coefficient, is rather sensitive to outliers. A small number of very high degree nodes who have mostly low-degree Friends can therefore easily reduce the coefficient, even when degree assortativity among the vast majority of nodes can be positive. Unfortunately, I have no means of verifying this hypothesis for the Cyworld data. In fact, I found a similar result for Hyves: when “one-way ties” are also included in the analysis, assortativity almost completely disappears. When I then restrict the analysis to edges between only nodes with degree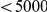, assortativity is again positive and similar to the result reported above.

**Table 3:** A comparison of three online social networks.

	Hyves	Facebook	Cyworld (2006)
Average degree	106.4	94.1	38.4
Fitted 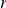	4.72	4.35	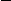
Clustering	0.18	0.16	0.16
Degree assortativity	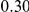	0.23	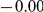

Facebook results as reported by [8]; Cyworld results as reported by [10].

## Discussion

This paper provides a first description of Hyves, a large online social network in the Netherlands. The results can be summarized as follows.

First, I find that although the membership of Hyves includes a considerable share of the Dutch population, it is not a demographically representative sample of this population. In particular, young people roughly between 10 and 25 years of age are overrepresented as a share of the population. The implication of this finding, even leaving aside possible discussions about the relation between online– and “offline” friendship, is that one should be very cautious to interpret the online friendship network as observed in Hyves as an approximation of *the* friendship network in the Netherlands. However, I also note that in the age groups of 10 to 25 years, Hyves seems to have a very large market share, to the extent that a vast majority of the people in this age group is a member. In combination with findings that show that specifically in this age group, online friendship does tend to reflect “offline friendship” [36], [37], complete online network data such as used in this study do seem to provide opportunities to study the structure of friendship networks among adolescents and (young) adults.

Second, I find that with regard to a number of key characteristics, the structure of the Hyves network follows the regularities found in other large complex networks. Specifically, I find that the network has a giant component containing virtually all members, has the properties of a “small world,” (clustering and small characteristic path lengths), and shows mild degree assortativity, indicative of a core-periphery structure. These findings suggest some implications for the way information or behavior can diffuse in such an online social network. The relative size of the giant component means that virtually everybody in the network can be reached, while the small-world structure means that information or behavior can spread relatively fast [19] in this network. Moreover, in the epidemiological literature, a core-periphery structure is believed to facilitate the spread of diseases, in the sense that that the core in such structures can serve as a “reservoir” for disease in which the disease keeps circulating, even though the disease may be restricted to this core [30]. Research on the diffusion on innovations, meanwhile, argues that the nodes in the core are often crucial for wide-spread adoption [38]. The implications of these mechanisms for the diffusion of information and behavior in the particular network structure of Hyves is a topic for further investigation.

Third, however, I also find that Hyves does not have a power law– or scale-free degree distribution. Thus, in this regard, the network deviates from what one might expect from the literature on large complex networks. Rather, I find a distribution akin to the multiscaling degree distributions also found in Facebook and Cyworld [8], [10]. Ahn et al. speculate that this finding suggests the presence of two types of members, who create links if different ways [10]. This conjecture is corroborated by my finding that high-degree members qualitatively differ from lower-degree nodes in terms of the clustering of their local networks. Moreover, in analyses not reported here, I found that high-degree members are somewhat older and more likely to be male, indicating that they also differ demographically. A mechanism that is likely to contribute to these difference is the possibility to become a “Goldmember” of Hyves. Other than regular members, Goldmembers pay a fee to use the service, in return for which they receive certain benefits, including the possibility to maintain more than 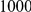 Friendships. This limit coincides with the value of the observed “cut” in the degree distribution and the sudden drop in clustering. However, it should be noted that the multi-scaling behavior of both the degree distribution and the clustering coefficient as a function of degree has also been observed in other online social networks in which no “institutionalized” distinction between members exists [8], [10], [39].

At the same time, I find that under the assumption that the distribution is created by a process involving both random growth and network-based link formation, the fitted parameter of this model indicates network is in the same range of randomness as some other social networks [25]. However, as compared to the earlier results on these networks, the fit of this model is relatively poor, which suggests that further effort at modeling this specific degree distribution is needed.

Fourth, I find that Hyves is remarkably similar to a number of other online friendship networks, as far as comparable results are available. Specifically, Hyves, Facebook, and Cyworld appear to have similar degree distributions, clustering, and, to a lesser extent, degree assortativity. It is worth stressing that these similarities occur despite large differences in network size and national contexts. Furthermore, I note that not only do all three networks deviate from the expectation of scale-free degree distributions, they seem to deviate *in similar same ways*. I take this as a first indication that online social (friendship) networks are governed by mechanisms that render them qualitatively different from other large complex networks. Because that preliminary conclusion is only based on three cases, however, more comparative research online social networks, as well as more detailed studies of the underlying mechanisms, would be needed.
